# Analysis of genetic control and QTL mapping of essential wheat grain quality traits in a recombinant inbred population

**DOI:** 10.1371/journal.pone.0200669

**Published:** 2019-03-06

**Authors:** Sonia Goel, Kalpana Singh, Balwant Singh, Sapna Grewal, Neeta Dwivedi, Abdulaziz A. Alqarawi, Elsayed Fathi Abd_Allah, Parvaiz Ahmad, N. K. Singh

**Affiliations:** 1 ICAR-National Research centre on Plant Biotechnology, New Delhi, India; 2 Water Technology Centre, Indian Agriculture Research Institute, New Delhi, India; 3 Plant Production Department, College of Food and Agricultural Sciences, King Saud University, Riyadh, Saudi Arabia; 4 Botany and Microbiology Department, College of Science, King Saud University, Riyadh, Saudi Arabia; 5 Department of Botany, S.P. College, Srinagar, Jammu and Kashmir, India; Institute of Genetics and Developmental Biology Chinese Academy of Sciences, CHINA

## Abstract

Wheat cultivars are genetically crossed to improve end-use quality for traits as per demands of baking industry and broad consumer preferences. The processing and baking qualities of bread wheat are influenced by a variety of genetic make-ups, environmental factors and their interactions. Two wheat cultivars, WL711 and C306, derived recombinant inbred lines (RILs) with a population of 206, were used for phenotyping of quality-related traits. The genetic analysis of quality traits showed considerable variation for measurable quality traits, with normal distribution and transgressive segregation across the years. From the 206 RILs, few RILs were found to be superior to those of the parental cultivars for key quality traits, indicating their potential use for the improvement of end-use quality and suggesting the probability of finding new alleles and allelic combinations from the RIL population. Mapping analysis identified 38 putative QTLs for 13 quality-related traits, with QTLs explaining 7.9–16.8% phenotypic variation spanning over 14 chromosomes, i.e., 1A, 1B, 1D, 2A, 2D, 3B, 3D, 4A, 4B, 4D, 5D, 6A, 7A and 7B. *In-silico* analysis based on homology to the annotated wheat genes present in database, identified six putative candidate genes within QTL for total grain protein content, *qGPC*.*1B*.*1* region. Major QTL regions for other quality traits such as TKW have been identified on 1B, 2A, and 7A chromosomes in the studied RIL population. This study revealed the importance of the combination of stable QTLs with region-specific QTLs for better phenotyping, and the QTLs presented in our study will be useful for the improvement of wheat grain and bread-making quality.

## Introduction

Recent research in wheat contributes to yield enhancement and disease resistance, but quality is lacking in today’s status quo. However, wide consumer demand has forced wheat breeders to focus on wheat quality improvement as per consumer preferences and industrial demands. Bread wheat (*Triticum aestivum* L.) is a globally accepted food crop and is consumed mainly in the form of baked products. The end-use quality of wheat is governed by a plethora of gene networks that are majorly affected by environmental conditions. Further, the end use property of wheat is measured by its seed quality and rheological traits such as Grain protein content (GPC), Sedimentation rate (SDS), Hectolitre weight (HW), 1000-kernel weight (TKW), Seed diameter (SD), Wet gluten content (WGC), Dry gluten content (DGC), Flour water absorption (FWA), Dough development time (DDT), Dough stability time (DST), Mixing tolerance index (MTI), Break down time (BDT) and Kernel hardness (KH). Quantitative traits loci (QTLs) for quality traits including GPC [1–3], KH [4,5], and dough quality traits, namely, MTI, mixing time, dough extensibility and dough tenacity [6, 7], have been mapped. Groos *et al*.(1) reported four QTLs for GPC on chromosomes 2A, 3A, 4D, and 7D.

Linkage mapping and subsequent QTL mapping is the prerequisite for applying a successful marker-assisted selection (MAS) programme for individual traits. Earlier, MAS was executed in hexaploid wheat for high GPC (*Gpc*-B1), which was mapped and introgressed from the wild tetraploid wheat *T*. *turgidum var*. *Dicoccoides* [8]. Further, the role of the QTLs (*Gpc-B1*) for increased GPC was confirmed in tetraploid and hexaploid wheat using near-isogenic lines (NILs) with distinct *Gpc-B1* alleles [9]. In additions, two independent studies conducted by Kumar *et al*. [10] and Tabbita *et al*. [11] showed that GPC was increased in Indian and Argentine hexaploid wheat carrying *Gpc-B1*. However, the pleotropic effect of the QTL *Gpc-B1* is associated with reduced grain size and grain yield that ultimately lead to a reduction in wheat production [11,12].

Dough rheological properties and KH strongly affect the end-use quality of wheat. Dough-making properties are often used as indicators of food baking quality. Dough strength and starch pasting characteristics are reported as quantitative traits; therefore, their expression is governed by multiple genes [13]. Presently, no specific bread-making quality trait-controlling genes have been identified that have direct associations with end-product quality. Nonetheless, a few QTLs for end-product quality traits have been reported [14]. Wheat quality is affected by temperature and humidity, but their effect is specific to developmental growth stages. Nuttall *et al*.[15] have reported that high temperatures during grain filling were responsible for reduced dough strength. Further, Cavanagh *et al*. [16] identified additional traits, such as the percentage of unextractable polymeric protein (%UPP) and dough strength, which were directly affected by temperature during the grain filling stage. The KH of wheat grain is a major determinant of food end-product quality. KH refers to the texture of the grain (caryopsis) that represents physical hardness or softness of the endosperm. KH is predominantly controlled by the Puroindoline (*Pin*) genes *Pin a* and *Pin b*,which are part of only the D sub-genome and are located on chromosome 5 at the Hardness (*Ha*) locus. Furthermore, different classes’ grain textures have been determined by unique allelic blends of *Pin* genes (*Pin a* and *Pinb*) in wheat, with diverse end-use characteristics [17]. The key role of the *Pin a* and *Pin b* genes is to determine the structure of the proteins in wheat grain, as well as the possible anti-microbial effects [18]. Therefore, to develop a variety with the desired KH, pronounced understanding of the allelic composition of *Pin* genes in a diverse set of germplasms is of the utmost importance for the selection of parental donors.

In the present study, a total population of 206 RILs was used for phenotyping of quality-related traits in three different locations in India, namely, Delhi, Karnal and Indore. The aim of the present research was to unravel the genetic factors controlling bread-making quality-related traits by mapping wheat population grown in three different environmental conditions through mapping of QTLs associated with these quality traits.

## Materials and methods

### Plant materials and experimental design

In the present study, a mapping population of 206 RILs (F_9:11_) was genotyped and evaluated for different quality traits.The RIL population was developed by crossing two wheat cultivars WL711 (S308/Chris/Kalyansona) and C306 (RGB/CSL3//2/C591/3/C217/N14//C281) [19]. WL711 is known for low end product quality traits while C306 is well known for its impact on good bread and cahapati making quality.The grain samples were taken from three independent field experiments conducted at Directorate of Wheat Research (DWR) in 2008, Karnal (76°09’E, 29°60’N; 228.6 M.S.L) (KL08), National Research Centre on Soybean (NRCS) Indore (75°50’E, 22°43’N; 529.9 M.S.L) in 2009 (IN09), and Division of Genetics, Indian Agriculture Research Institute, New Delhi (77°12’E, 28°40’N; 228.6 M.S.L), India in 2010 (DL10). These three regions are geographically located in the traditional wheat agro-ecosystems. RILs, along with parents, were sown in three environments in a randomized complete block design (RCBD) pattern in the field with three replications per experiment. Sowing was done in a plot containing 3 rows that were 1.5 m long; each row was equally spaced by 25 cm and in each row, a total of 30 seeds were planted. RILs were sown during mid November and harvesting was done in April at DL10 and KL08, while at IN09, they were sown in early November and harvesting was done in early March.

### Quality traits analysis

RIL grain samples collected from each experimental location were analysed in the same year at Cereal Quality Laboratory, Division of Genetics, Indian Agriculture Research Institute, New Delhi, India (S1 Table). There were three replicates used from each experiment for each quality traits samples were hand-cleaned and air-aspirated to remove foreign material and shrivelled kernels. The estimation of GPC was done by near-infrared reflectance (NIR) (RACI-CCD, 2010) using a NIR instrument (Foss 6500, FOSS NIR Systems, Inc., Laurel, MD) [20]. Estimated sedimentation volume, represented in height (mm) of the sediment measured during the SDS sedimentation test, was estimated as gluten strength [21]. Wheat flour of 206 RILs and the two parental genotypes (WL711 and C306) used for quality analysis was produced by a Cyclotec Mill (Tecator AB, Sweden) fitted with a 1 mm sieve. Five flour quality traits, namely, BDT, DDT, DST, FWA, and MTI were recorded by a Farinograph (Brabender, Germany) according to AACC 2000 [20].

Clean samples of 20 g seeds with grain moisture content ranging between 10% and 11% were used for the analysis of KH, TKW and SD using the Single Kernel Characterization system (SKCS) 4100 (Perten Instruments, Australia) with the AACC method (2000). HW was measured as the volume of grain per unit. Further, grain protein gluten was measured as wet and dry gluten using Glutomatic 2200 (Perten Instruments) according to the AACC method (2000).

### Statistical analysis of the traits

Statistical and genetic analysis for quality traits was performed by GenStat14 [22]. The analysis was conducted in two stages while taking account of experimental design factors, first spatial analysis [23], to find the best linear unbiased estimates (BLUEs). Analysis of variance (ANOVA) was conducted for all traits separately for estimating variance components for evaluation of the significance of genotypes and trial effects and their interactions in the WL711/C306 RIL population. ANOVA was done using three factor factorial analysis of the statistical programme MSTAT-C, version 1.41, Michigan State University, USA.

The broad sense heritability (*h*_B_^2^) value was calculated for each trait across environments as

h^2^ = σ^2^_g_/(σ^2^_g_+ σ^2^_gxe_/e)

whereσ^2^_g =_ [MS_RIL_- MS_RILxe_)/e], σ^2^_gxe_ = MS_RILxe_,

Wheree is the number of environments, MS is the mean square and x is the sign of multiplication.

### Genetic analysis of the traits

The information regarding the genotyping of the RIL population and linkage map was given in Shukla *et al*. [24].

### *In-silico* identification of genes within QTL region

Total genes present within underlying QTLs were identified utilising NCBI blast to reference chromosome from wheat genome sequence. Markers flanking (gmw413 and cfd65) to the QTL *qGPC*.*1B*.*1* was blast to 2A wheat chromosome sequence and genomic sequence of the QTL region was downloaded. Annotated CDS present within the QTL region was selected from the wheat genome annotated CDS present in EMBL database (ftp://ftp.ensemblgenomes.org/pub/plants/release-42/fasta/triticum_aestivum). Function of the genes was predicted using blast2go tool [25]. Genes having more than 70% of the similarity were selected.

## Results

### Phenotypic data and correlation analysis

Experiments were conducted at three locations in three different years. Performance of both the parents was observed along with the RIL populations. WL711 showed a low quality score at KL08 compared to that of the other locations; however, C306 showed better performance for traits at the same location and years (Table 1). Measurable phenotypic variation was observed among both the parents for SDS, TKW, WGC, DGC, FWA, DST, MTI, BDT and KH. All quality-related traits significantly differed among the RILs and exhibited transgressive segregation (Table 1). A combined ANOVA was performed over all trials which indicated statistically significant main effects for genotypes (G), trials (T), GxT interactions for quality traits (Table 2). Variance due to GxT interaction was substantially lower than variation due to genotype for all the traits. GPC and HW showed high broad sense heritability, while TKW and KH showed moderate heritability (Table 2). A highly significant positive correlation was recorded between GPC, WGC, DGC and FWA; between SDS,WGC, DGC, DDT and DST; between DDT,BDT, and KH; and between DST and BDT. Highly significant but negative correlations were recorded between GPC and TKW; between WGC and DST; and between MTI, DST, and BDT (Table 3).

**Table 1:** Quality parameters in parents and RIL population derived from WL711/C306.

Traits	Trials	Parental lines		RIL population		
		WL711	C306	Min	Max	Mean
GPC	DL09	11.4 ± 0.71	14.7 ± 0.16	10.5	18.5	13.9 ± 0.33
	KL08	11.2 ± 0.37	13.9 ± 0.24	9.8	16.2	11.5 ± 0.11
	IN09	11.9 ± 0.25	15.6 ± 0.21	11.2	19.9	15.9 ± 0.41
SDS	DL09	45.8 ± 2.9	56.5 ± 1.7	24	75.8	46.0 ± 2.2
	KL08	42.4 ± 1.5	53.5 ± 1.1	21.6	71.5	43.0 ± 1.2
	IN09	48.2 ± 1.8	57.8 ± 1.3	23.7	73.5	49.0 ± 3.2
HW	DL09	77.5 ± 3.6	73.8 ± 4.3	55.5	83.8	78.7 ± 2.0
	KL08	72.1 ± 2.4	74.1 ± 2.7	54.9	81.2	74.2 ± 1.3
	IN09	79.5 ± 4.1	76.5 ± 3.9	59.3	87.6	79.4 ± 4.8
TKW	DL09	35.6 ± 1.4	41.5 ± 1.3	27.4	52.7	41.1 ± 1.9
	KL08	33.3 ± 2.1	44.2 ± 1.5	22.2	55.3	39.5 ± 1.2
	IN09	37.2 ± 2.7	45.5 ± 1.8	25.7	58.1	46.2 ± 1.7
SD	DL09	2.75 ± 0.18	2.98 ± 0.25	2.12	3.5	3.03 ± 1.0
	KL08	2.22 ± 0.14	3.18 ± 0.19	2.63	2.5	2.92 ± 1.4
	IN09	2.64 ± 0.19	2.76 ± 0.32	2.81	3.9	3.66 ± 1.7
WGC	DL09	28.5 ± 1.1	36.8 ± 1.6	23.5	48	35.5 ± 1.5
	KL08	24.9 ± 1.5	37.2 ± 1.3	21.2	45.9	35.8 ± 1.8
	IN09	26.2 ± 1.9	39.1 ± 1.4	25.7	47.3	33.3 ± 2.1
DGC	DL09	9.5 ± 0.6	12.5 ± 0.8	7	14.8	11.2 ± 1.2
	KL08	8.9 ± 0.2	13.2 ± 0.3	5.5	13.4	10.7 ± 1.3
	IN09	9.8 ± 0.8	13.9 ± 0.6	8.3	14.9	11.9 ± 1.7
FWA	DL09	58.5 ± 1.3	64.2 ± 2.4	54.6	68.5	60.8 ± 2.7
	KL08	56.9 ± 1.2	66.1 ± 2.2	54.8	66.2	61.1 ± 2.8
	IN09	59.1 ± 1.7	66.9 ± 2.8	55.2	65.9	60.9 ± 2.9
DDT	DL09	3.8 ± 0.4	5.4 ± 0.8	2.1	8.5	4.5 ± 0.6
	KL08	3.3 ± 0.1	5.3 ± 0.5	2.6	8.2	4.2 ± 0.2
	IN09	3.5 ± 0.8	5.8 ± 0.6	2.2	8.8	4.7 ± 0.8
DST	DL09	2.8 ± 0.2	8.5 ± 0.5	1.5	10.8	4.0 ± 0.3
	KL08	2.4 ± 0.3	8.3 ± 0.6	1.3	10.3	4.3 ± 0.6
	IN09	2.7 ± 0.8	8.2 ± 0.8	1.8	10.6	4.8 ± 0.5
MTI	DL09	105.4 ± 6.8	45.0 ± 3.2	25	151.6	80.5 ± 2.8
	KL08	102.1 ± 5.2	43.0 ± 4.1	22.7	153.1	80.1 ± 2.7
	IN09	107.7 ± 6.5	48.0 ± 3.6	25.9	154.9	82.5 ± 2.1
BDT	DL09	4.2 ± 0.5	11.5 ± 0.8	2.2	14.5	7.5 ± 0.6
	KL08	3.1 ± 0.2	12.8 ± 0.3	2.9	12.6	7.1 ± 1.2
	IN09	3.8 ± 0.6	11.9 ± 0.1	2.4	15.8	7.7 ± 0.8
KH	DL09	89 ±2.5	60 ± 3.5	99	68	83.5 ± 4.0
	KL08	78 ±1.4	62 ± 3.8	85	62	82.2 ± 2.6
	IN09	87 ±2.2	59 ± 3.2	92	66	86.7 ± 4.3

Grain protein content (GPC, %), Sedimentation rate (SDS), Hectolitre weight (HW, g), 1000-kernel weight (TKW, Seed diameter (SD), Wet gluten content, WGC, %), Dry gluten content (DGC, %), Flour water absorption (FWA, %), Dough development time (DDT, min), Dough stability time (DST, min), Mixing tolerance index (MTI, F.U), Break down time (min) (BDT), Kernel hardness (KH), KL08 = Directorate of Wheat Research (DWR) in 2008, Karnal, IN09 = National Research Centre on Soybean (NRCS) Indore in 2009, and DL10 = Division of Genetics, Indian Agriculture Research Institute, New Delhi, India in 2010

**Table 2:** Analysis of varianceof quality traits of wheat RIL population across Delhi, Karnal and Indore trials.

Trait code/ Sources of variation	Gf	Mean Square Value												
		GPC	SDS	HW	TKW	SD	WGC	DGC	FWA	DDT	DST	MTI	BDT	KH
**Replicates**	2	2.4^ns^	3.7^ns^	4.2^ns^	3.5^ns^	0.34^ns^	3.2^ns^	1.7^ns^	4.2^ns^	0.76^ns^	0.31^ns^	3.6^ns^	0.79	5.8^ns^
**Genotypes (G)**	205	34.1*	65.8*	88.2*	43.6*	10.3*	36.8*	28.5*	67.4*	12.5*	9.5*	42.1*	9.5*	81.5*
**Trials (T)**	2	2476*	3575*	5423	3654*	102*	3265*	1856*	4563*	134*	105*	4245*	102*	5634*
**G x T**	409	45.2*	68.4*	89.6*	64.7*	14.7*	53.7*	32.7	69.6*	15.3*	12.6*	56.2*	13.2*	89.5*
**Error**	617	34.8	68.5	93.2	66.3	16.8	59.6	36.4	72.5	17.5	15.1	57.9	16.3	96.4
**CV**		4.8	3.2	5.2	2.9	7.6	8.5	7.2	2.4	6.1	6.4	5.2	0.5	5.2
**CD**		5.7	5.2	6.7	3.8	8.4	10.4	8.1	4.6	9.5	9.3	6.8	1.5	7.4
***h***^**2**^		0.61	0.52	0.76	0.63	0.59	0.40	0.49	0.55	0.61	0.58	0.61	0.33	0.60

Grain protein content (GPC, %), Sedimentation rate (SDS), Hectolitre weight (HW, g), 1000-kernel weight (TKW, g), Moisture content (MC), Seed diameter (SD), Wet gluten content (WGC, %), Dry gluten content (DGC, %), Flour water absorption (FWA, %), Dough development time (DDT, min), Dough stability time (DST, min), Mixing tolerance index (MTI, F.U), Break down time (min) (BDT), Kernel hardness (KH), CV- Coefficient of variation, CD- Critical differences *h*^2^_B_ Genotype mean heritability of all trials.

*Statistically significant (p≤0.05)

ns- Non significant

**Table 3:** Correlation analysis of quality related traits in RIL population derived from WL711/C306.

Traits	GPC	SDS	HW	TKW	SD	WGC	DGC	FWA	DDT	DST	MTI	BDT	KH
**GPC**	1												
**SDS**	0.478*	1											
**HW**	-0.354*	-0.214*	1										
**TKW**	-0.687**	-0.178*	-0.485*	1									
**SD**	-0.342*	-0.173*	-0.043	-0.128*	1								
**WGC**	0.795**	0.318**	-0.167*	0.126*	0.043	1							
**DGC**	0.832**	0.353**	-0.268*	0.121*	0.165*	0.825**	1						
**FWA**	0.432**	-0.234*	-0.143*	0.034	-0.321*	-0.143*	0.032	1					
**DDT**	-0.085	0.312**	0.031^ns^	0.021	0.012	0.856**	-0.162	0.003	1				
**DST**	-0.041	0.483**	0.023	0.124*	0.114*	-0.243**	-0.173	0.008	0.881**	1			
**MTI**	-0.108	-0.323*	-0.284*	-0.542*	-0.013	-0.092^ns^	-0.101*	-0.343*	-0.583**	-0.777**	1		
**BDT**	0.095	0.014	0.053	0.154*	0.003	-0.226**	-0.155	0.021	0.822**	0.877**	-0.767**	1	
**KH**	-0.234*	-0.143*	0.043	0.143*	0.04	0.184*	0.029	0.02	0.824**	0.329*	0.036	0.019	1

Grain protein content (GPC, %), Sedimentation rate (SDS), Hectolitre weight (HW, g), 1000-kernel weight (TKW, g), Moisture content (MC), Seed diameter (SD), Wet gluten content (WGC, %), Dry gluten content (DGC, %), Flour water absorption (FWA, %), Dough development time (DDT, min), Dough stability time (DST, min), Mixing tolerance index (MTI, F.U), Break down time (min) (BDT), Kernel hardness (KH)

*Statistically significant (p≤0.05)

**Statistically significant (p≤0.01)

ns- Non significant

### QTLs for bread-making traits

**Fig 1:**
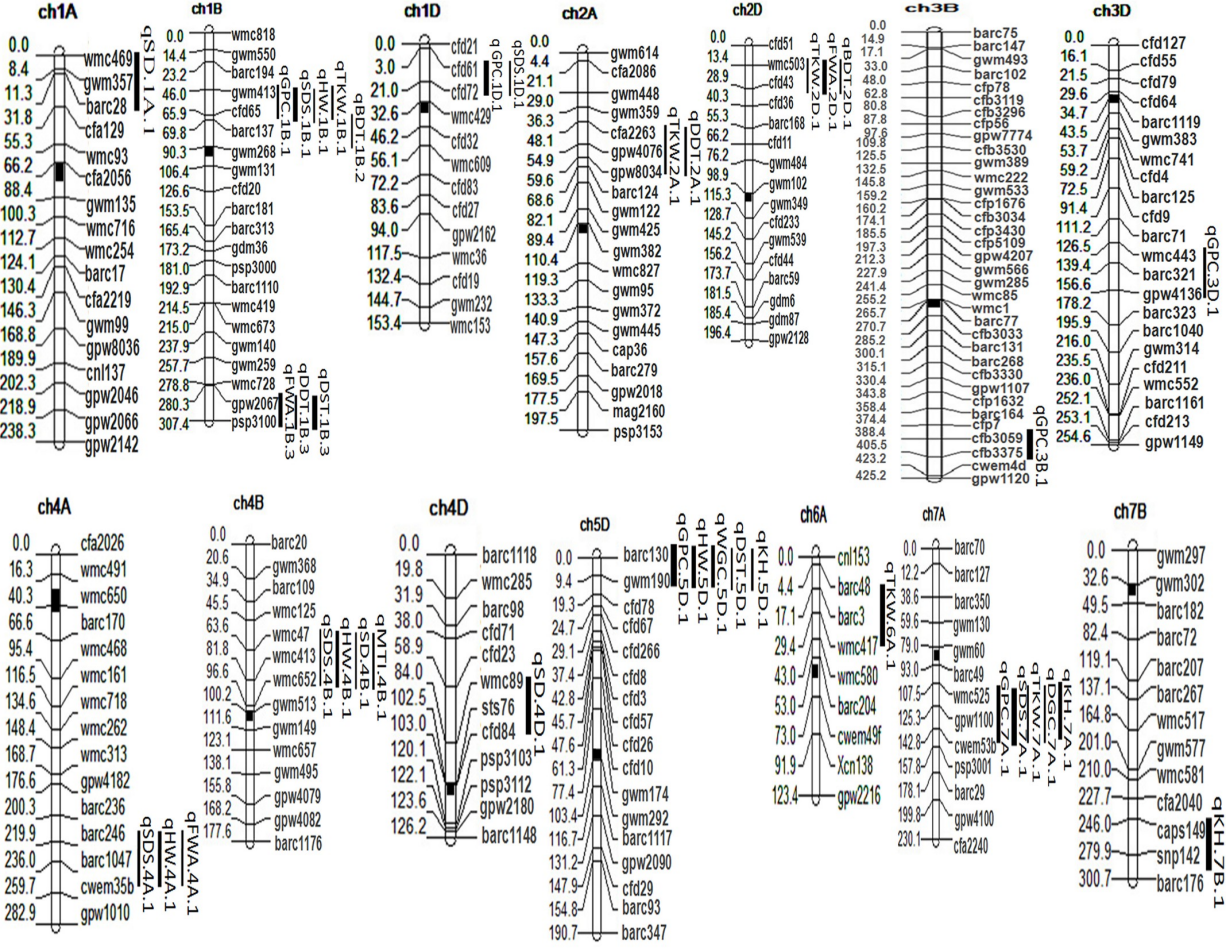
Quantitative trait loci (QTLs) for quality traits in WL711/C306 wheat RIL population. The vertical bars indicate the QTL confidence intervals. Map distances (cM) are shown on the left side of each chromosome.

Overall, 38 putative QTLs related to 13 bread-making quality traits were reported, explaining 7.9% to 16.8% phenotypic variance (PV) (Table 4,). The QTLs were dispersed on 14 chromosomes of all three (A, B and D) genome types, i.e., 1A, 2A, 4A, 6A, 7A,1B, 3B, 4B, 7B, 1D, 2D, 3D, 4D and 5D (Fig 1). Six QTLs were identified for GPC on chromosomes 1B, 1D, 3B, 3D, 5D and 7A, explaining 9.8% to15.8% of PV. Alleles were contributed by WL71l at two QTLs (*qGPC*.*3D*.*1* and *qGPC*.*7A*.*1*) and by C306 at four QTLs (Table 4). The strongest effect for GPC (11.9), with 15.8% PV, was located on *qGPC*.*5D*.*1* with the allele being contributed by C306. *qGPC*.*5D*.*1* was found to be co-located with the QTLs, explaining HW, WGC, DST and KH. Another major QTL for GPC was *qGPC*.*7A*.*1* which explained 13.9% of PV, and the allele was contributed by WL711. This QTL was observed to show co-location with QTLs responsible for SDS, TKW, DGC and KH.

**Table 4:** Major and minor QTLs for quality traits identified by composite interval mapping analysis using QTL cartographer software in 206 RIL population derived from WL711/C306.

Traits^1^	QTL	Trials	Marker Interval	Position cM	LOD value	*a* Effect^2^	R^2^(%)^3^
GPC	*qGPC*.*1B*.*1*	DL09, IN09, KL08	gwm413—cfd65	43.7	3.1	-4.7	10.3
	*q GPC*.*1D*.*1*	DL09, KL08	cfd61—cfd72	2.6	4.3	-3.6	12.4
	*qGPC*.*3B*.*1*	DL09	cfb3059—cfb3375	317.1	5.7	-2.6	12.8
	*qGPC*.*3D*.*1*	IN09, KL08	wmc443—gpw4136	139.4	3.6	4.8	9.8
	*qGPC*.*5D*.*1*	DL09, KL08, IN09	barc130—gwm190	0.01	7.8	-11.9	15.8
	*qGPC*.*7A*.*1*	KL08	wmc525—cwem53b	115.5	3.1	6.9	13.9
SDS	*qSDS*.*1B*.*1*	DL09, IN09	gwm413—cfd65	43.7	4.5	34.6	11.6
	*qSDS*.*1D*.*1*	IN09	cfd61—cfd72	2.5	3.3	-2.3	10.1
	*qSDS*.*4A*.*1*	KL08, IN09	barc246—cwem35b	238.1	4.1	12.6	9.6
	*qSDS*.*4B*.*1*	DL09, KL08	wmc47—wmc652	63.6	3.1	9.6	9.0
	*qSDS*.*7A*.*1*	IN09	wmc525—cwem53b	115.5	5.7	-44.7	16.8
HW	*qHW*.*5D*.*1*	DL09, IN09	barc130—gwm190	0.01	7.4	-46.1	14.7
	*qHW*.*4A*.*1*	KL08, IN09	barc246—cwem35b	238.1	3.8	5.7	10.5
	*qHW*.*1B*.*1*	DL09, KL08	gwm413—cfd65	43.7	3.1	13.6	9.5
	*qHW*.*4B*.*1*	DL09, IN09	wmc47—wmc652	63.6	3.6	4.7	11.6
TKW	*qTKW*.*1B*.*1*	DL09	gwm413—cfd65	43.7	3.5	26.2	10.6
	*qTKW*.*2A*.*1*	DL09, KL08	cfa2263—gpw8034	54.9	3.6	28.6	10.3
	*qTKW*.*2D*.*1*	KL08, IN09	wmc503—cfd43	28.9	4.3	-37.9	10.8
	*qTKW*.*6A*.*1*	DL09, KL08, IN09	barc48—wmc417	23.5	4.2	-41.6	14.1
	*qTKW*.*7A*.*1*	DL09, KL08, IN09	wmc525—cwem53b	115.5	6.7	-42.6	15.8
SD	*qSD*.*1A*.*1*	DL09	wmc469 -barc28	8.4	3.7	-2.1	12.6
	*qSD*.*4B*.*1*	DL09, KL08, IN09	wmc47—wmc652	63.6	2.9	1.2	7.9
	*qSD*.*4D*.*1*	KL08	wmc89 -cfd84	102.5	5.8	-2.6	14.7
WGC	*qWGC*.*5D*.*1*	IN09, KL08	barc130—gwm190	0.01	4.6	-26.1	13.5
DGC	*qDGC*.*7A*.*1*	DL09	wmc525—cwem53b	115.5	3.7	-10.6	11.8
FWA	*qFWA*.*1B*.*3*	IN09, KL08	gpw2067—psp3100	292.3	4.2	63.2	14.1
	*qFWA*.*2D*.*1*	DL09. IN09	wmc503—cfd43	28.9	4.3	-36.8	15.3
	*qFWA*.*4A*.*1*	DL09, KL08	barc246—cwem35b	238.1	3.1	16.8	8.7
DDT	*qDDT*.*1B*.*3*	DL09, KL08, IN09	gpw2067—psp3100	292.3	4.1	2.6	11.4
	*qDDT*.*2A*.*1*	DL09, KL08	cfa2263—gpw8034	54.9	3.6	1.8	10.3
DST	*qDST*.*1B*.*3*	IN09, KL08	gpw2067—psp3100	292.3	4.6	-5.7	14.1
	*qDST*.*5D*.*1*	IN09, KL08	barc130—gwm190	0.01	3.2	-3.8	10.4
MTI	*qMTI*.*4B*.*1*	DL09	wmc47—wmc652	63.6	3.1	54.6	8.7
BDT	*qBDT*.*1B*.*2*	DL09, KL08, IN09	cfd65—gwm268	77.8	3.5	12.5	9.4
	*qBDT*.*2D*.*1*	DL09. IN09	wmc503—cfd43	28.9	4.3	11.5	10.8
KH	*qKH*.*5D*.*1*	DL09, KL08	barc130—gwm190	0.01	3.8	46.8	9.5
	*qKH*.*7A*.*1*	DL09, KL08, IN09	wmc525—cwem53b	115.5	5.4	-65.4	14.6
	*qKH*.*7B*.*1*	DL09, KL08	caps149—snp142	256	4.1	-55.4	13.6

^1^Traits: Grain protein content (GPC, %), Sedimentation rate (SDS), Hectolitre weight (HW, g), 1000-kernel weight (TGW, g), Seed diameter (SD), Wet gluten content (WGC, %), Dry gluten content (DGC, %), Flour water absorption (FWA, %), Dough development time (DDT, min), Dough stability time (DST, min), Mixing tolerance index (MTI, F.U), Break down time (BDT, min), Kernel hardness (KH)

^2, 3^additive main effects, R2 (*a*) % phenotypic variation explained by *a*effects. A positive value of the additive main effects (*a*) indicates that WL711 contributes allele to increase the trait, and a negative value means that C306 provides allele to increase the trait.

Five QTLs associated with SDS sedimentation were identified on chromosomes 1B, 1D, 4A, 4B and 7A, explaining 9.0% to16.8% of PV. Out of the five QTLs for SDS sedimentation, three QTLs (*qSDS*.*1B*.*1*, *qSDS*.*4A*.*1* and *qSDS*.*4B*.*1*) came from WL711 and two (*qSDS*.*1D1* and *qSDS*.*7A*.*1*) were from C306 (Table 4). The largest favourable effect on SDS sedimentation (44.7) was associated with *qSDS*.*7A*.*1* and explained 16.8% of PV; the allele was contributed by C306. Another high-impact QTL for SDS was *qSDS*.*1B*.*1*, which had a major additive effect (34.6) that was identified on chromosome 1B, explaining 11.6% of PV.

Four QTLs for HW were identified on chromosomes 1B, 4A, 4B and 5D, explaining 9.5% to 14.7% of PV; three QTLs came from WL711, and one came from C306 (Table 4). The QTL *qHW*.*5D*.*1* had an additive effect (46.1), explained 14.7% of PV, and co-localized with QTLs for GPC, WGC, DST and HW. For TKW, a total of 5 QTLs were reported on chromosomes 1B, 2A, 2D, 6A and 7A, explaining 10.3% to15.8% of PV. A major QTL for TKW, *qTKW*.*7A*.*1*, was identified on 7A, with the allele coming from C306 and explaining 15.8% of PV. This QTL was co-located with QTLs for GPC, SDS, DGC and KH.

Three QTLs were identified for SD on chromosomes 1A, 4B and 4D, explaining 7.9% to14.7% of PV. Another QTL for SD, *qSD*.*4D*.*1*, was identified on 4D, explaining 14.7% of PV; this positive allele was contributed by C306 (Table 4). Another major QTL responsible for WGC was identified on chromosome 5D, explaining 13.5% of PV, and this positive allele was contributed by C306. A major QTL for DGC, *qDGC*.*7A*.*1*, was identified on chromosome 7A and explained 11.8% of PV; this positive allele was contributed by C306 and was found to be co-located with QTLs for GPC, TKW and KH. A total of 3 QTLs for FWA were identified on chromosomes 1B, 2D and 4A, explaining 8.7% to15.3% of PV. A major QTL, *qFWA*.*2D*.*1*, was identified on 2D, explaining 15.3% of PV, with this positive allele contributed by C306. This QTL co-located with QTLs for TKW and BDT.Two QTLs for DDT were identified on chromosomes 1B and 2A. Another QTL for DDT was identified on 2A, *qDDT*.*2A*.*1*, which explained 10.3% of PV, and this positive allele was contributed by WL711 and was co-located with TKW. Two QTLs, namely, *qDST*.*1B*.*3* and *qDST*.*5D*.*1*, that were identified for DST were located on chromosomes 1B and 5D and explained 14.1% and 10.4% of PV, respectively. A major QTL for DST, *qDST*.*1B*.*3* on chromosome 1B, explained 14.1% of PV, and the allele was contributed by C306.

A minor QTL responsible for MTI was identified on 4B, explaining 8.7% of PV. Two QTLs for BDT were identified on chromosomes 1B (*qBDT*.*1B*.*2*) and 2D (*qBDT*.*2D*.*1*), explaining 9.4 and 10.8% of PV, respectively (Table 4). Two major QTLs and a minor QTL for KH were identified on chromosomes on 7A, 7B and 5D, explaining 9.5 to 14.6% of PV. A major QTL for KH, *qKH*.*7A*.*1* on chromosome 7A, explained 14.6% of PV, and this positive allele was contributed by C306.

### QTL x environment interactions and epistatic QTL

The effects of the QTL x environment interactions (QE) for quality-related traits were recorded and listed in Table 5. From the measured quality traits, two QQ interactions were detected for GPC and TKW. In addition, a few genomic regions identified in this study showed QE, QQ and QQE interactions, and their effects were less noticeable than the main additive effects (a). These results indicated that the additive effects were more significant than the epistatic effects in the studied quality traits. Epistatic QTLs showed QTL x QTL (QQ) and QTL x QTL x environment (QQE) interaction.

**Table 5:** Etpistatic QTLs and QTL x QTL x environment interaction for quality related traits identified by two locus analysis using QTL Network software in 206 RILs derived from WL711/C306.

Traits^a^	QTL_i^b^	Interval_i	QTL_j	Interval_j	QQ^c^	QQE^d^	R2%	
							QQ^e^	QQ E^f^
GPC	*qGPC*.*1B*.*1*	gwm413—cfd65	*q GPC*.*1D*.*1*	cfd61—cfd72	0.21	0.16	0.08	0.03
TKW	*qTKW*.*1B*.*1*	gwm413—cfd65	*qTKW*.*2D*.*1*	wmc503—cfd43	-0.42	-0.13	0.08	0.05

A positive value means that the parent-type effect is greater than the recombinant-type effect

A negative value means that the parent-type effect is less than the recombinant-type effect

^a^ GPC- grain protein content, TKW- thousand kernel weight

^b^QTL_i and QTL_j are a pair of QTL involved in epistasis

^c^QQ, the epistatic main effect

^d^QQE, the epistasis x environment interaction effects

^e^ R2 (QQ) %, Phenotypic variation explained by QQ effects

^f^ R2 (QQE) %, Phenotypic variation explained by QQE effects

### Gene identification within QTLregion

The genomic region within the flanking markers of QTL *qGPC1B*.*1* was retrieved from the NCBI genome database. *In-silico* analysis showed total 346 genes were found within this QTLs region. Out of 346, 110 genes showed more that 70% functional similarity with the existing protein in database. Among these 110 genes, 50 were enzymes, 7 transcription factor, 4 transporters, 7 ribosomal protein, 6 chloroplast, 5 motochondial subunits encoded by genome, 4 receptors and 27 were belongs to different function (S2 Table). Further analysis based on homology to the annotated wheat genes present in database showed that only six genes namely PGKY_Phosphoglycerate kinase, cytosolic, CBP2_Serine carboxypeptidase2, PALY_Phenylalanine ammonia-lyase, HBP1C_Transcription factor HBP-1b (c1), MT1_Metallothionein-like protein 1 and UBC2_Ubiquitin-conjugating enzyme belongs to Triticum aestivum (Table 6).

**Table 6:** Genes senquence name and genomic position of identified genes within the flanking markers of QTL *qGPC1B*.*1*based on homology to the annotated wheat genes present in the database.

SeqName	Genomic position	Description
TraesCS1B02G104200.1	114686573	PGKY_Phosphoglycerate kinase, cytosolic
TraesCS1B02G104500.1	115278975	CBP2_Serine carboxypeptidase 2
TraesCS1B02G122800.1	148413936	PALY_Phenylalanine ammonia-lyase
TraesCS1B02G127400.3	156565523	HBP1C_Transcription factor HBP-1b(c1)
TraesCS1B02G135800.1	173121735	MT1_Metallothionein-like protein 1
TraesCS1B02G140300.1	185548114	UBC2_Ubiquitin-conjugating enzyme

## Discussion

### Phenotypic and genotypic variation in the parents and the RILs

Growing genotypes under well-adapted conditions with strong phenotypic expression can lead to overestimation of the genetic component, which could be avoided by including contrasting environments and seasons in which observations are made. In accordance with this notion, the experimental materials consisting of a population of 206 RILs that was developed from the cross WL711/ C306 were grown under three environmental conditions. A total of 38 QTLs were identified through CIM for thirteen quality-related traits across environments. Continuous phenotypic variation and transgressive segregation for all the traits observed in the RIL population revealed the quantitative inheritance of these traits. Further, both the parents contributed beneficial allele for quality traits strengthened usefulness of this population for QTL analysis and genetic interaction analysis between the alleles.

### Genetic locus for quality traits GPC, TKW and KH

Increased GPC is a focus area of current wheat quality breeding programmes. Parent C306 and the RILs showed a significantly high mean GPC (above 15%) in the IN09 environment, where RILs were exposed to heat between post-anthesis and the grain filling stage. These results were in agreement with Maphosa *et al*. [26]. GPC showed a low value (below 12%) in the DL10 and KL08 experiments, when crops experienced cool and moist conditions. Li *et al*. [27] indicated that total GPC is linked to temperature and low humidity. A negative correlation between GPC and TKW was recorded in this population, which was reported in previous studies as well [28]. The QTLs related to GPC were reported earlier on the regions of several chromosomes, showing several loci controlling wheat GPC; those studies also suggested very fewer differences in GPC in the parental line, but QTLs were still detected [29, 30]. In the present study, QTL analysis for GPC revealed six QTLs with PV ranging from 9.8–15.8% located on six different chromosomes, i.e., 1B, 1D, 3B, 3D, 5D and 7A. The chromosomes 3B and 7A were earlier also explored for the GPC content [31]. Although, the difference in protein content between the parents was lower, transgressive segregants were observed for GPC. These transgressive segregants for high GPC might be due to minor genes segregating in the population and the different GPC-controlling alleles in the parents, confirming the suitability of this population for QTL analysis for GPC [32]. A set of epistatic QTLs showed weak additive × additive × environment effects (AAE), andthe interactions suggested that the additive effects played an important role in wheat GPC. In this study, QTLs for GPC and SDS were mapped near the *Glu-D1* region which is present on chromosome 1D. Similar results were observed in other studies as well [33, 34]. In fact, the *Glu-D1*gene that codes HMW subunits (2+12 and5+10) was also found to affect the protein quality in a ChSh population [35]. Furthermore, another wheat protein, triticin, which is encoded by *Tri–D1*, was reported to positively affect wheat dough bread-making quality, which was also present on the short arm of chromosome 1D [36]. The other two QTLs for GPC, on chromosomes 3B and 5D, had larger effects and can be used for further genetic improvement.

TKW is one of the important yield components. Selection of TKW directly increases the grain yield [37]. Its correlation with quality parameters has been reported [38]. Selection for quality traits alone will not improve this trait. A pronounced and significant variation for TKW suggested several genes with major and minor effects that were involved in the phenotypic expression of this trait. TKW was controlled by 5 QTLs identified in our study, which were present on the chromosomes 1B, 2A, 2D, 6A and 7A.Sun *et al*. [39] also identified seven QTL regions on chromosomes 2A, 2D, 3B, 4A, 5D, 6A, 6B, and 7B in RIL population. In addition, Reif *et al*. [40] identified 12 putative QTLs on chromosomes 1A, 3A, 5A, 7A, 1B, 3B, 6B, 1D, 3D, 4D and 7D in a RIL population. In these studies, only one QTL(6B) was found similar, which suggested that many genes govern the trait TKW. Of the eight QTLs identified by Sun *et al*. [38] only two QTLs i. e. 2D and 6A, shared chromosomal location in the present study. Wheat chromosome 7A was earlier also endorsed for the study of QTLs for different agronomic traits and also for TKW as similar to our study [41]. Recently, MAS was used for the transfer of three garin weight QTL *QGw*.*ccsu-1A*.*2*, *QGw*.*ccsu-1A*.*3* and *QGw*.*ccsu-1B*.*1* identified from NILs derived from Raj3765 and K9107 [42]. In this study, one epistatic QTL was identified with negative Additive × Environment (AE) or AAE interactions, which showed that an additive effect responsible for the main genetic variance of TKW.

KH played a major role in determining quality of bread wheat and end use properties. Additionally, the *Ha* locus is mainly known for affecting grain hardness in wheat. Several QTLs for KH that are distributed on all twenty-one wheat chromosomes except for 3D and 6A have been reported in different mapping populations [43]. Both parents contributed favourable alleles for KH, which confirmed the quantitative nature of the trait [44].

### Identification of gene-rich regions/ QTL clusters

In wheat, associations of qualitatively inherited genes together represent gene-rich regions form the hot spots of recombination. QTL are usually spread over all the chromosomes, but clusters of QTLs in certain chromosomal regions have been observed. QTLs affecting several traits are common and may be due to pleiotropy or close linkage [34]. Since most of the QTL hotspots in this study were located in the short and long arm of the chromosomes, QTL co-location of yield QTLs has also been identified previously in wheat [1, 37]. Similarly, 5 QTLs were mapped on 5D,5 QTLs on 7B and 4 QTLs on 1B, and some of them showed stability across the environments, which also suggested that the two QTL clusters might have pleiotropic effects. It is likely that the clusters represent similar gene/protein content. Several linked markers in the clusters suggest the usefulness of these markers for marker-assisted breeding of these QTLs to enhance the end-product quality of wheat.

## Conclusions

Overall, 38 QTLs for 13 end product quality traits were mapped, explaining 7.9 (*qSDS*.*4B*.*1*) to 16.8% (*qSDS*.*7A*.*1*) of PV detected on total 14 chromosomesi.e.,1(ABD), 2(A, D), 3(B,D), 4(ABD), 5D, 6A, 7A and 7B. The additive effect was found to be positive in 17 QTLs, contributed by WL711 while, 21 were negative and contributed by C306. Eight QTLs for three major quality traits affecting the bread-making quality, namely, SDS (5), DST (2) and DGC (1), were identified, with 9.6 to 16.8% PV. For SDS, five of the three alleles were contributed by WL711, and for DST and DGC, both were contributed by C306..For GPC, six QTLs were reported on chromosome 1B, 1D, 3B, 3D, 5D and 7A, showing 9.8–15.8% of PV for the trait, with positive alleles coming from WL71l at two QTLs (*qGPC*.*3D*.*1* and *qGPC*.*7A*.*1*) and from C306 at four QTLs. The strongest effect for GPC (11.9), with 15.8% PV, was located on *qGPC*.*5D*.*1*, with the positive allele being contributed by C306. Six putative candidate genes have been identified by *In-silico* analysis of QTL *qGPC*.*1B*.*1* region based on homology to the annotated wheat genes present in the database. This study revealed the importance of the combination of stable QTLs with region-specific QTLs for better phenotyping, and the QTLs presented in our study will be useful in MAS efforts after validation for the improvement of wheat grain and bread-making quality.

## Supporting information

S1 Table Mean value of the measured traits data of a 206 RILs population along with parents recored from three independent experiments conducted at three different locations (KL08, IN09, DL10) in an independent year. (XLSX)

S2 Table List of genes present within flanking markers of QTL *qGPC1B*.*1* were identifed form EMBL database and function was pridicted based on homology by Blast2go tool. (XLSX)

S1 Data **Table A:** Quality parameters in parents and RIL population
**Table B:** Major and minor QTLs for quality traits.
(XLSX)
